# Implementing the WHO AWaRe antibiotic book guidance in lower-resource settings: the case of the Lao PDR

**DOI:** 10.1093/jacamr/dlae004

**Published:** 2024-01-22

**Authors:** Vilada Chansamouth, Phouthavanh Inlorkham, Bounxou Keohavong, Khonsavath Bellingham, H Rogier van Doorn, Mayfong Mayxay, Paul N Newton, Paul Turner, Nicholas P J Day, Elizabeth A Ashley

**Affiliations:** Lao-Oxford-Mahosot Hospital-Wellcome Trust Research Unit (LOMWRU), Mahosot Hospital, Vientiane, Lao PDR; Microbiology Laboratory, Mahosot Hospital, Vientiane, Lao PDR; Centre for Tropical Medicine & Global Health, Nuffield Department of Medicine, University of Oxford, Oxford, UK; Food and Drug Department, Ministry of Health, Vientiane, Lao PDR; Food and Drug Department, Ministry of Health, Vientiane, Lao PDR; Lao-Oxford-Mahosot Hospital-Wellcome Trust Research Unit (LOMWRU), Mahosot Hospital, Vientiane, Lao PDR; Nuffield Department of Medicine, Oxford University Clinical Research Unit, Hanoi, Viet Nam; Lao-Oxford-Mahosot Hospital-Wellcome Trust Research Unit (LOMWRU), Mahosot Hospital, Vientiane, Lao PDR; Centre for Tropical Medicine & Global Health, Nuffield Department of Medicine, University of Oxford, Oxford, UK; Institute of Research and Education Development (IRED), University of Health Sciences, Ministry of Health, Vientiane, Lao PDR; Centre for Tropical Medicine & Global Health, Nuffield Department of Medicine, University of Oxford, Oxford, UK; Faculty of Infectious and Tropical Diseases, London School of Hygiene and Tropical Medicine, London, UK; Centre for Tropical Medicine & Global Health, Nuffield Department of Medicine, University of Oxford, Oxford, UK; Cambodia Oxford Medical Research Unit, Angkor Hospital for Children, Siem Reap, Cambodia; Centre for Tropical Medicine & Global Health, Nuffield Department of Medicine, University of Oxford, Oxford, UK; Mahidol Oxford Tropical Medicine Research Unit (MORU), Faculty of Tropical Medicine, Mahidol University, Bangkok, Thailand; Lao-Oxford-Mahosot Hospital-Wellcome Trust Research Unit (LOMWRU), Mahosot Hospital, Vientiane, Lao PDR; Centre for Tropical Medicine & Global Health, Nuffield Department of Medicine, University of Oxford, Oxford, UK

## Abstract

In 2022, WHO released the WHO AWaRe (Access, Watch, Reserve) antibiotic book to promote the rational use of antibiotics. Here, we review the AWaRe antibiotic book from the perspective of implementation in low-resource settings, using the Lao PDR (Laos) as a case study. Not all recommendations in the AWaRe antibiotic book match the epidemiology of infectious diseases and antimicrobial susceptibility patterns in Laos and other low- and middle-income countries (LMICs), e.g. melioidosis, rickettsial disease and leptospirosis are common causes of sepsis and febrile illness in Laos but do not feature in the AWaRe book. Conversely, some infectious diseases like *Clostridioides difficile*-associated diarrhoea are in the AWaRe antibiotic book but rarely considered in Laos with no diagnostic tests available. Only 29/39 antibiotics in the AWaRe book are available in Laos, with no Reserve group antimicrobials available. The AWaRe book stimulates countries such as Laos to consider alternative diagnoses and include additional antimicrobials in the national essential medicines list (NEML). However, it should be updated to include regional important pathogens that are not included. Comprehensive antibiotic use guidelines alone might not assure appropriate use or control overuse of antibiotics. Access to antibiotics is challenging in low-resource settings in terms of unavailability in the country (low demand or small market size), patchy access, especially for those living in remote areas, and unaffordability. All these systemic factors can contribute to inappropriate use of antibiotics. Improved access to antibiotics, strengthening diagnostic capacity and promoting antibiotic stewardship should be combined.

## Introduction

Antimicrobial resistance (AMR) is widely considered to be one of the top health threats worldwide. Although AMR occurs naturally, evidence shows that misuse and overuse of antimicrobials accelerate the development of acquired resistance.^1,2^ In many low- and middle-income countries (LMICs), there are fewer antibiotics available than in high-income countries, and it can be difficult to access them, particularly in remote areas. It has been argued that increasing access to antibiotics requires more complex strategies to improve quality of care and appropriate use of antibiotics.^3–5^ Comprehensive antimicrobial prescribing guidelines with proper training and appropriate implementation approaches have been proposed as an option to improve the rational use of antibiotics.^6^

In 2022, WHO released the first WHO AWaRe (Access, Watch, Reserve) antibiotic book, an adjunct to the Essential Medicines List, with the aim of providing ‘…short, clinical guidance on the management of common infections’ in hospital and primary healthcare settings.^7^ This global guideline includes 39 antibiotics and covers 34 common infection presentations in adults and children, focusing on empirical treatment. Among the 39 antibiotics, 20 (51.2%) are in the Access antibiotic group, 11 (28.2%) in the Watch group and 8 (20.5%) in the Reserve group.^7^ The WHO AWaRe Antibiotic Book is not intended to replace existing local or national prescribing guidelines. However, several countries still do not have these,^8^ and so it provides a useful template for adapting to the local context. For countries with guidelines, it can act as a reference standard against which to benchmark. There are expected to be challenges to implementing the guidelines in some LMICs, e.g. due to lack of laboratory diagnostics, human resources and access.^9^

The first comprehensive Lao national antimicrobial prescribing guidelines were made available in 2021. These guidelines were developed based on the current local antimicrobial susceptibility patterns and antimicrobial use (AMU) situation in Laos, existing local treatment guidelines and international recommendations.^10,11^ Currently, the guidelines only recommend antibiotics that are registered in the country, with two exceptions: vancomycin (for treatment of MRSA bacteraemia only) and nitrofurantoin (for ESBL-producing *Escherichia coli* urinary tract infection, due to high rates of resistance to all other antibiotics). Here we review the WHO AWaRe antibiotic book from the perspective of implementation in low-resource settings, using Lao PDR (Laos) as a case study. Our framework of evaluation considers the epidemiology of infectious diseases and AMR in Laos, the current situation with regard to antimicrobial use, availability and access, and practical considerations such as feasibility, including systemic barriers at hospital or policy level.

## Infectious disease and AMR epidemiology in Laos

Laos is a land-linked country in the WHO Western Pacific region, sharing borders with Vietnam, Thailand, China, Myanmar and Cambodia. Antimicrobial-treatable infectious diseases such as melioidosis, rickettsial disease and leptospirosis are well documented in Laos.^12–16^ In a study of the epidemiology of febrile illness in Laos between 2008 and 2010, dengue fever was the leading cause of febrile illness, diagnosed in 8% (156/1927) of patients, followed by scrub typhus in 7% (122/1871), Japanese encephalitis virus in 6% (112/1924), leptospirosis in 6% (109/1934) and other bloodstream infections (BSIs) at 2% (43/1938).^14^ HIV prevalence in Laos is 0.3%,^17^ and malaria incidence has declined dramatically over the last two decades, now being restricted to a small number of southern provinces.^18^ Diagnostic microbiology is mainly available in a few hospitals in Vientiane, the capital city, although services have been expanded to provincial hospitals in the last 2 years, with financial support from a UK Fleming Fund country grant.^19^ Data generated by the Microbiology Laboratory of Mahosot Hospital, a large central hospital in Vientiane in 2022, showed that among 7913 blood cultures taken, the most common pathogen causing bacteraemia in patients of all ages was *Burkholderia pseudomallei*, in 134 (1.7%), followed by *E. coli* in 111 (1.4%) and *Staphylococcus aureus* in 69 (0.9%) [Lao-Oxford-Mahosot Hospital-Wellcome Trust Research Unit (LOMWRU), unpublished data]. The main AMR concern in Laos currently is an increase in numbers of BSIs caused by ESBL-producing *E. coli.* Rates of ESBL-*E. coli* isolated from blood increased from 7% (95% CI 0.9–25) of all *E. coli* isolates in 2004 to 35% (95% CI 24–46) in 2016^15^ and reached 58.5% (95% CI 49–68) in 2022. Rates of MRSA bacteraemia have also risen, currently around 26% (95% CI 16–38) in 2022 (LOMWRU, unpublished data).

The WHO AWaRe book covers 34 common infection presentations. The majority of them are included in the Lao antimicrobial prescribing guidelines.^10,11^ However, treatment recommendations for the most common infections in Laos such as melioidosis, rickettsial diseases and leptospirosis are not included in the WHO AWaRe book. As a result, for some syndromes, recommendations do not match the epidemiology of infectious diseases and antimicrobial susceptibility patterns in Laos. For example, Lao antimicrobial prescribing guidelines suggest ceftazidime (to cover melioidosis) combined with amikacin (to cover ESBL-producing *E. coli*) as a primary choice for empirical treatment of septic shock, and meropenem as an alternative choice. Bacterial meningitis is another example; causes of CNS infections in Laos from 2003 to 2011 showed that zoonotic diseases (rickettsial diseases and leptospirosis) were common, causing 7.5% (80/1065) of suspected CNS infections in Laos, after Japanese encephalitis virus at 8.8% (94/1065).^20^ Therefore, our local guidelines suggest ceftriaxone combined with doxycycline for suspected bacterial CNS infections (Table 1). A recent publication from Varghese *et al*.^22^ suggested that the combination of IV doxycycline and azithromycin was more effective than doxycycline or azithromycin alone for severe rickettsial diseases. This should be taken into a consideration to update Lao national prescribing guidelines for severe infections. However, parenteral doxycycline and azithromycin are not currently available in Laos.

**Table 1.** Recommended antibiotic treatment for selected severe clinical presentations in adults in Lao antimicrobial prescribing guidelines versus the WHO AWaRe book

Lao antimicrobial prescribing guidelines—adults^10^	WHO AWaRe book—adults^7^	Comments
Empirical treatment for suspected bacterial meningitis	Empirical treatment for suspected bacterial meningitis	Doxycycline is included to cover zoonotic diseases such as rickettsia and leptospirosis, which are commonly found as a cause of CNS infections in Laos.^20^
* *Ceftriaxone 2 g, IV every 12 h PLUS doxycycline 200 mg, PO first dose, then 100 mg every 12 h OR (if severe penicillin allergy) chloramphenicol 1–2 g, IV every 6 h. Add ampicillin or amoxicillin if risk factors for *L. monocytogenes* infection; add aciclovir 10 mg/kg, IV every 8 h if suspected meningoencephalitis.	* *First choice: cefotaxime 2 g, IV every 6 h OR ceftriaxone 2 g, IV every 12 h. Add ampicillin or amoxicillin if risk factors for *L. monocytogenes* infection. * *Second choice: amoxicillin 2 g, IV every 4 h OR ampicillin 2 g, IV every 4 h OR benzylpenicillin 4 million IU (2.4 g), IV every 4 h OR chloramphenicol 1 g, IV every 6 h.	
Empirical treatment for septic shock	Empirical treatment for septic shock	Melioidosis is a leading cause of death from sepsis in Laos and treatment is with ceftazidime and not ceftriaxone.^12^ More than 50% of invasive *E. coli* infections are ESBL positive and resistant to gentamicin (LOMWRU, unpublished data) so Lao guidelines recommend amikacin first line; however, it is only available in Vientiane capital. Clarithromycin IV, piperacillin/tazobactam and vancomycin are not in the NEML in Laos.^21^
* *Ceftazidime 2 g, IV every 8 h PLUS amikacin 25–30 mg/kg/day, IV every 24 h OR gentamicin 7 mg/kg/day, IV every 24 h. Add metronidazole if suspect abdominal sepsis.	* *Cefotaxime 2 g, IV every 6 h OR ceftriaxone 2 g, IV every 12 h PLUS: * *Unknown origin: amikacin 15 mg/kg, IV every 24 h OR gentamicin 5 mg/kg, IV every 24 h * *Lower respiratory tract infection: clarithromycin 500 mg, IV every 12 h * *Intra-abdominal infection: metronidazole 500 mg, IV every 8 h or piperacillin/tazobactam 4g + 500 mg, IV every 6 h * *Urinary tract infection: amikacin 15 mg, IV every 24 h * *Skin and soft tissue infection: metronidazole 500 mg, IV every 8 h (add vancomycin 15–20 mg/kg, IV every 12 h if MRSA suspected).	

PO, by mouth (per os).

Conversely, some topics in the WHO AWaRe book are not included in Lao prescribing guidelines, such as *Clostridioides difficile* infection, acute diverticulitis and infectious uveitis, which could be reconsidered. Although only five patients with *C. difficile* infection have ever been reported in Laos in a research study, it may be underreported since *C. difficile* diagnosis is not routinely available.^23^

## Antibiotic use in Laos

According to the first hospital antimicrobial use point-prevalence survey in Laos, the use of antimicrobials among admitted patients in five provincial hospitals and one central referral hospital was ∼70% from 2017 to 2020. Antibiotics from the WHO Access group accounted for 50% of prescriptions, which was below the WHO target (60%), while 49% were from the Watch group and 0.8% were ‘not recommended’ [ceftriaxone/sulbactam in 30/33 (91%) and cefoperazone/sulbactam in 3/33 (9%)].^24^ Use was much higher than the reported global average from 53 countries, which was 34%,^25^ and surrounding countries (53% from Thailand in 2021,^26^ 63.4% from Myanmar in 2019^27^ and 67.4% from Vietnam in 2008^28^). Chansamouth *et al*.^24^ also revealed that ceftriaxone was heavily used at all surveyed hospitals, accounting for 39.6% (2596/6555) of all prescriptions. Ceftriaxone made up 38.4% of prescriptions for treating infections and 50.6% (720/1424) of all surgical prophylaxis prescriptions. The use of antibiotics in outpatient departments or for milder infections at one central and five provincial hospitals in Laos was 25% (3106/13 325), including the use of those in the Access group (66%; 2334/3526) and the Watch group (34%; 1192/3526) during 2021 and 2022.^29^ Data on the use of antibiotics in primary healthcare settings in Laos are limited. However, a recent study of more than 25 000 consultations in health centres in southern Laos found that only around 10% result in an antibiotic prescription (Koukeo Phommasone, LOMWRU, Mahosot Hospital, Laos, personal communication, 2023). A systematic review and meta-analysis of 48 studies from 27 LMICs to determine the use of antibiotics in primary healthcare showed a much higher proportion of antibiotic use, at 52% (95% CI 51–53), with the proportion of inappropriate use of antibiotics ranging from 8% to 100%.^30^ Improving antimicrobial stewardship (AMS) is under strategic objective four out of five objectives of the national strategic plan on AMR in Lao PDR 2019–23. The national AMS programme has been piloted in a central hospital in Laos to monitor and promote the rational use of antimicrobials; however, AMS activities in other hospitals are still at a very early stage.

### Availability and access to antibiotics in Laos

The WHO AWaRe book includes 39 antibiotics. The most recent national essential medicines list (NEML) registered with the Lao Food and Drug Department (FDD), Lao Ministry of Health in 2019, included 29 antibiotics (either oral or parenteral or both administration routes; excluding anti-TB and anti-leprosy medicines). Of these 29 agents, 15 (52%) were in the WHO Access group and 14 (48%) were in the WHO Watch group. No medicine from the Reserve group has been registered in Laos (Table 2).^21^ While, on the one hand, this prevents inappropriate use of the last-resort antibiotics, it also means that a small number of patients with XDR infections are not able to access the medicines. The only option currently is for family members or physicians to source these medicines from neighbouring countries on a case-by-case basis.

**Table 2.** List of recommended antibiotics from the WHO AWaRe book that are not in the Lao NEML (2019) in Laos

Group	Antibiotics
Access	Cefazolin, clarithromycin (injection), clindamycin, nitrofurantoin, spectinomycin, sulfamethoxazole/trimethoprim (injection), trimethoprim
Watch	Cefuroxime, piperacillin/tazobactam, vancomycin
Reserve (none are currently in the Lao NEML)	Aztreonam, carumonam, cefiderocol, ceftaroline fosamil, ceftazidime/avibactam, ceftobiprole medocaril, ceftolozane/tazobactam, colistin (injection), colistin (oral), dalbavancin, dalfopristin/quinupristin, daptomycin, eravacycline, faropenem, fosfomycin (injection), iclaprim, imipenem/cilastatin/relebactam, lefamulin, linezolid, meropenem/vaborbactam, minocycline (injection), omadacycline, oritavancin, plazomicin, polymyxin B (injection), polymyxin B (oral), tedizolid, telavancin, tigecycline

Comparing the national guidelines in Laos to the WHO AWaRe antibiotic book highlights antimicrobials that would be useful to introduce in Laos, as well as some instances where there is a more practical alternative already in use, or where there are barriers to following the AWaRe recommendations. Mahosot Hospital Microbiology Laboratory reported 172 *E. coli* isolates from urine specimens in 2022, of which 61.6% (106) were ESBL-producing *E. coli*. Of 105 tested, 104 (99%) were susceptible to nitrofurantoin (LOMWRU, unpublished data), which is recommended in the WHO AWaRe antibiotic book. However, nitrofurantoin is not available in Laos. Access to and use of nitrofurantoin might facilitate reduction in the use of Watch group antibiotics and may both decrease the number of hospital admissions with ESBL-producing *E. coli* BSI and slow down the spread of ESBL-producing bacteria by reducing the use of ceftriaxone in Laos.

Cefazolin, a first-generation cephalosporin, is the first-choice antibiotic for surgical prophylaxis of clean procedures, or cefazolin combined with metronidazole for contaminated procedures or bowel surgery.^31^ The AWaRe book mostly recommends cefazolin for surgical prophylaxis.^7^ Cefazolin is not in the registered NEML for Laos.^21^ Based on antibiotic availability in Laos, Lao antimicrobial prescribing guidelines recommend amoxicillin/clavulanic acid or ceftriaxone, with or without metronidazole, for surgical prophylaxis.^10,11^ Repeated point-prevalence surveys from 2017 to 2020 have shown that the use of antibiotics in surgical departments accounted for one-third of all antimicrobial prescriptions in Lao hospitals.^24^ Most antibiotic use for surgical prophylaxis lasted more than 1 day, which is not recommended.^15^ Cefixime, an oral third-generation cephalosporin antibiotic in the Watch group, was among the top five antibiotics (185/3520; 5%) prescribed in outpatient departments in Laos during 2021 and 2022 in six hospitals across the country. The main purpose of the prescription was for treatment of presumed sexually transmitted infections (61/185; 33%). However, it was also used for respiratory infection (45/185; 24%), urinary tract infections (28/185; 15%) and pharyngitis (27/185; 14%), for which Access antibiotics can be prescribed.^29^ Even though guidelines are available, achieving the appropriate use of antibiotics can still be challenging.

The AWaRe book cautiously recommends meropenem as a secondary choice for sepsis/septic shock, bacterial meningitis, febrile neutropenia and some gastrointestinal tract infections. Meropenem was included in the Lao NEML in 2019; Lao antimicrobial prescribing guidelines recommend meropenem as an alternative choice for septic shock, melioidosis and infection with laboratory confirmation of ESBL-producing pathogens. Meropenem was among the top 10 antibiotics prescribed among admitted patients in six hospitals in Laos from 2021 to 2022,^29^ and carbapenem resistance has been reported in Laos since 2015.^32^ Vancomycin has been introduced in some hospitals in Vientiane, for patients diagnosed/suspected with MRSA bacteraemia. The use of vancomycin in Laos is challenging as a result of unpredictable supply, cost and the fact that it is not included in the NEML. Lack of therapeutic drug monitoring (TDM) could lead to toxicity or the risk of it being at subtherapeutic concentrations.^33^ Clindamycin is proposed in the AWaRe book for treatment of MRSA skin and soft tissue infections, which is inexpensive (USD 0.12/tablet in Laos), but the majority of isolates in Laos currently are not susceptible to this agent, which is also not widely available. Of 127 *S. aureus* isolates from all submitted pus specimens to the Microbiology Laboratory, Mahosot Hospital for culture from August to November 2023, 61 (48%) were MRSA. Of these MRSA isolates, 60 (98%) were resistant to clindamycin but only 1 (1.6%) was resistant to sulfamethoxazole/trimethoprim (LOMWRU, unpublished data). Current practice in Laos is to use sulfamethoxazole/trimethoprim, which is available, inexpensive and still highly susceptible.

## Practical considerations

Recommendations (choice, dose and duration) on antimicrobial prescribing differ country by country.^8^ Access to up-to-date standard antibiotic prescribing guidelines is needed for all health workers to prescribe appropriately and to control the spread of AMR. However, in practice, in low-resource settings such as Laos, comprehensive prescribing guidelines alone might not be sufficient. Unlike in high-income countries, where the use of broad-spectrum antibiotics like meropenem is usually discussed with infectious disease specialists before prescribing, there are currently no restrictive AMS policies in Laos.

Poor antibiotic access is a global issue and lack of economic incentives have been shown to be the major reason for pharmaceutical companies not producing or marketing them.^34^ This is likely to be worse in small LMICs with low demand and small market size, like Laos, leading to a perception of no return on investment to register or market them in these countries.^35^ Unavailable antibiotics in the country or uneven availability with people living in remote areas unable to access certain antibiotics, or high cost of antibiotics could lead to poor prescribing and poor patient outcomes. On the other hand, some broad-spectrum antibiotics that are not included in the Lao NEML are circulating in some larger hospitals,^24,21^ risking faster spread of AMR. However, this might also reflect the need for certain antibiotics for the country and updating of the NEML (in Laos it is updated every 3 years and the last version was in 2019) based on local scientific evidence and international recommendations. Limited access to microbiology diagnosis and poor understanding of diagnostic stewardship are other challenges to following guidelines and prescribing appropriately. Monitoring the use of antibiotics and strengthening the drugs and therapeutics committee in each hospital may help to overcome this. An up-to-date NEML that reflects prescribing guidelines, adherence to prescribing recommendations by clinicians, and prioritization of the issue by health governance bodies will likely increase the demand for the right kind of antibiotics, providing an incentive for pharmaceutical companies to market them.

The WHO AWaRe book is very welcome as a global antibiotic use guideline, which incorporates useful information to diagnose infectious diseases and laboratory considerations. LMICs developing national guidelines need to adapt them to the local epidemiology of infectious diseases and antimicrobial susceptibility patterns. For example, melioidosis is a common cause of sepsis in Laos and other Southeast Asian countries but has been largely overlooked in the WHO AWaRe book, as have rickettsial infections. In other settings with a high burden of HIV, TB has been described as a common sepsis presentation.^36^ Incorporating some of these regional variations in infectious disease epidemiology in future editions of the WHO AWaRe book would be very useful for countries and make them truly global guidelines. Countries submitting all antimicrobial susceptibility data to WHO GLASS rather than just selected bug–drug combinations could facilitate this. At the same time, the lack of diagnostic capacity in many LMICs makes creating locally appropriate guidelines challenging. There is evidence that guidelines frequently do not take local antimicrobial susceptibility into account.^37^ In addition, lack of access to several antibiotics is a barrier to implementing the guidelines. In a small country like Laos, there might not be much incentive for manufacturers to try and register antimicrobials.

### Conclusions

Recommendations in the AWaRe book provide a useful reminder to health workers and policymakers in countries such as Laos to consider expanding access to key antimicrobials and promote their appropriate use, as well as expanding infectious disease diagnosis and antimicrobial stewardship efforts, and making sure that the antimicrobial prescribing guidelines are updated regularly. However, they should adapt them to local infectious disease epidemiology and antimicrobial susceptibility data. Having easy access to antibiotics and comprehensive antibiotic use guidelines does not guarantee appropriate use or prevent overuse if the diagnostic capacity is lacking and antibiotic stewardship is not promoted.
